# Nanosized SnO_2_ Prepared by Electrospinning: Influence of the Polymer on Both Morphology and Microstructure

**DOI:** 10.3390/polym13060977

**Published:** 2021-03-23

**Authors:** Alberto Rubin Pedrazzo, Claudio Cecone, Sara Morandi, Maela Manzoli, Pierangiola Bracco, Marco Zanetti

**Affiliations:** 1 Department of Chemistry, University of Torino, Via P. Giuria 7, NIS and INSTM Reference Centres University of Torino, 10125 Torino, Italy; alberto.rubinpedrazzo@unito.it (A.R.P.); claudio.cecone@unito.it (C.C.); sara.morandi@unito.it (S.M.); pierangiola.bracco@unito.it (P.B.); 2 Department of Drug Science and Technology, NIS and INSTM Reference Centres University of Torino, Via Giuria 9, 10125 Torino, Italy; maela.manzoli@unito.it; 3 ICxT Centre, University of Torino, Lungo Dora Siena 100, 10153 Torino, Italy

**Keywords:** electrospinning, oxide, tin oxide, nanofibers, nanoparticles

## Abstract

An electrospinning (ES) procedure of polymeric solutions containing metal oxide precursors, followed by thermal treatments, was exploited to obtain SnO_2_ nanofibers. Attention was focused on the effect of different templating polymers (polyvinyl pyrrolidone (PVP), polyethylene oxide (PEO) and polyvinyl acetate (PVAc)) on the morphologies and particle size distributions of SnO_2_. We demonstrated that with different polymers, the final oxide’s morphology and crystallite size change. Defined fibers, with homogeneous diameter, were obtained with each polymer, but, after calcination, the morphology of the oxide changes, leading to fibers, “flakes” or “sphere-shaped” particles when PVP, PEO or PVAc were used, respectively, as evidenced by SEM images. Data from HR-TEM and XRD measurements confirm that SnO_2_ samples consist of crystalline cassiterite, with small mean particle dimensions calculated by Debye–Scherrer equation, i.e., 30, 11 and 25 nm with PVP, PEO and PVAc, respectively. TEM measurements put in evidence lower average particle sizes and for SnO_2_ obtained with PEO average size of 8.5 nm with a standard deviation of ±4.9 nm was evidenced. By applying different calcination temperatures on fiber mat obtained by the same polymer, i.e., PEO, the influence of polymer not only on the final shape of the oxide particles but also on the crystallite size was definitively demonstrated.

## 1. Introduction

In the last 40 years in the ultra-sensible sensors field, there has been a growing interest and a wide diffusion of metal oxide semiconductors (MOS). MOS are suitable materials for sensors with high-efficiency, fast response, stability and simple fabrication [1,2]. Tin oxide (SnO_2_) is a versatile metal oxide that combines low electric resistance with high transparency in the visible range: these peculiarities are exploited, besides the sensing field, in a wide range of applications, for example, as an electrode in solar cells and in displays [3,4,5]. SnO_2_ can be used as catalytic support for metals and, combined with vanadium oxides, for catalyzed oxidation of aromatic compounds and in the synthesis of carboxylic acids and acid anhydrides [6,7]. In solar cells, photocatalysis and gas sensing applications, SnO_2_ activity is strongly related to the specific surface area that is related to particle/crystallite sizes. To improve the properties of SnO_2_ and, consequentially, to improve the efficiency of SnO_2-_based devices, research in the last years has moved to nanosized structures with controllable crystalline phases. Oxide nanoparticles and nanofibers have been recently largely studied: the organization in this particular morphology is interesting for all those applications where it is requested high specific surface area [8,9].

There are many examples of synthetic methods for obtaining ceramic nanoparticles, such as thermal and physical deposition and hydro/solvothermal processes [3,5].

Over the last few years, the procedure of electrospinning (ES) of polymeric solutions containing precursors of ceramics, followed by thermal treatment, has been adopted for achieving ceramic nanofibers [10,11,12,13,14,15,16,17]. ES is a relatively simple and versatile technique to obtain both micro and nanofibers of polymers accumulated on a mat, usually showing high porosity and high specific surface area [18,19,20,21]. In a typical ES process, a polymer solution is injected through a small nozzle under the application of a strong electric field (in the order of kV/cm); the presence of electrostatic charges on the surface of the solution drop leads to the formation of a jet attracted from a collector at the opposite of the electric field. During the flight from the nozzle to the collector, the solvent evaporates, and the polymer precipitates in the form of a thin fiber.

Adding oxide precursors to the ES solution allows the formation of polymeric fibers containing a certain amount of precursor. A subsequent thermal treatment, above the thermal degradation of the polymer, will ablate the polymer, transforming at the same time the precursor in the respective oxide. In this way, it is possible to obtain oxide fibers of different diameters and morphology using the polymer as a templating agent.

There are many examples in the literature of SnO_2_ nanofibers or nanoparticles obtained with ES and subsequent thermal treatment [13,22,23,24]: nonetheless, in all these works, the polymer involved in the electrospinning process is always considered as a mere physical template and not as a variable capable of influencing morphological and textural properties of the oxide. In the present work, nanostructured SnO_2_ was obtained from three different templating polymers via electrospinning and subsequent calcination of the electrospun fiber mat. The polymers used as templates were poly(vinylpyrrolidone) (PVP), poly-(ethylene oxide) (PEO) and poly-(vinyl acetate) (PVAc), widely used for electrospinning, common and with different structures and thermal behaviors [19,20,25,26,27,28,29,30]. The obtained SnO_2_ nanoparticles were characterized by scanning electronic microscopy, high-resolution transmission electron microscopy (HR-TEM) and by X-ray diffraction (XRD), evidencing a possible influence of the polymer.

## 2. Experimental

### 2.1. Materials

The polymers used in this comparison were the following, all purchased from Sigma-Aldrich (Darmstadt, Germany): poly(vinylpyrrolidone) (PVP, Mw 1,300,000), poly-(ethylene oxide) (PEO, Mw 600,000), poly-(vinyl acetate) (PVAc, Mw 500,000). The SnO_2_ precursor was tin (II) 2-ethylhexanoate (SnEt, also purchased from Sigma-Aldrich) with a purity grade of 94.4%. *N,N*-dimethylformamide (DMF) was purchased from Sigma-Aldrich with a purity grade ≥ of 98.8%.

### 2.2. Preparation of the Samples

For a significant comparison, even if PVP is easily soluble in water, we worked using anhydrous DMF for the preparation of all solutions. Additionally, dimethylformamide permits a better dispersion of the organic Sn precursor. Different polymer solutions were tested before using DMF, starting from water and water/organic solvents mixtures (water/ethanol, water/acetone, water/DMF). Unfortunately, the precursor precipitates in water (as SnO_2_ particles), and even with the addition of acetic acid and/or 2-ethylhexanoic acid, which can help to shift the equilibrium of the hydrolysis reaction, it is impossible to obtain a stable solution that can be electrospun.

The solutions (Table 1 for details) were prepared in closed vials, adding the precursor only after complete solubilization of the polymer (POL) in DMF. The solubilization was carried out at room temperature (within 30 min) for all the samples except for the polymer PEO, heated at 70 °C and sonicated for 1.5 h.

The solutions were loaded into a syringe (BD 3 mL equipped with a BD PrecisionGlide needle with 18 gauge, purchased from Sigma-Aldrich) for the electrospinning process: the needle of the syringe was connected to a high voltage (DC generator, GLASSMAN High Voltage, Pulborough, West Sussex, UK) set at a voltage of 30 kV. The collector for the electrospun fibers was a rotating drum (cylinder-shaped, length: 120 mm and diameter 80 mm made) covered with a paper sheet. The solution was ejected from the syringe with a controlled feeding rate, set at 20 µL/min. The working distance (from the tip of the needle and the collector) for all preparation was 20 cm, and all the electrospinning process was conducted under controlled temperature (room temperature, RT) and humidity. After the electrospinning process, the obtained fiber mats were calcined in an oven (from Nabertherm, work range 30–3000 °C) to eliminate the polymeric matrix and convert the precursor in the correspondent oxide. Further details of the calcination, such as temperature, will be discussed in the next section.

As reported in Table 1, the ratio between precursor and polymer was maintained constant, though the concentrations of polymer in DMF are different, depending on the appropriate viscosity needed for obtaining regular fibers.

### 2.3. Characterization Techniques

The morphological characterization was performed by scanning electron microscopy (SEM), using a Leica Stereoscan 410 microscope by Oxford Instruments (Abingdon, UK) (W filament) operating at 20 kV. For each electrospun sample, the distribution of the fiber diameters was obtained by counting a statistically representative number of fibers, and the mean diameter (d_m_) was calculated as d_m_ = Σd_i_n_i_/Σn_i_, where n_i_ represents the number of fibers of diameter d_i_.

Moreover, high-resolution transmission electron microscopy (HR-TEM) was employed to achieve further morphological and structural information of the SnO_2_ samples obtained after calcination of the fibers with a side entry Jeol (Akishima, Tokyo, Japan) JEM 3010 UHR (300 kV, LaB_6_ filament).

In the former case, the samples were gold-sputtered prior to the examination, whereas in the latter one, the synthesized samples were deposited on a Cu grid, coated with a porous carbon film. All digital micrographs were acquired by an UltraSscan 1000 camera, and the images were processed by Gatan digital micrograph (Pleasanton, CA, USA). To obtain the SnO_2_ particle size distribution, a statistically representative number of crystallites was counted for each measured sample (>500 and >250 counted nanoparticles for SnO_2_(PEO) and SnO_2_(PVP), respectively). The mean particle diameter (d_m_) was calculated as d_m_ = Σd_i_n_i_/Σn_i_, where n_i_ represents the number of particles of diameter d_i_.

To have information on the structure of the SnO_2_ obtained after thermal treatment, X-ray diffraction (XRD) analysis was carried out on a PW3050/60 X’Pert PRO MPD (PANalytical, Malvern, UK) (X-ray source is a PW3373/10 LFF with Cu Anode, λ = 0.541 Å). To determine the suitable temperature for the degradation of the polymer and of the SnO_2_ organic precursor, thermo-gravimetric analysis (TGA) of the electrospun samples was performed by TGA Q500 from TA Instruments (New Castle, DE, USA), with the following program: 10 °C/min from RT to 800 °C with a 100 mL/min airflow.

## 3. Results and Discussion

### 3.1. Electrospinning of Polymer/Precursor 

The aim of this work is a comparison of the SnO_2_ obtained from the calcination of fiber mats made via ES of different polymer solutions containing tin (II) 2-ethylhexanoate. Therefore, the first step was the obtaining of regular fibers, with comparable size, diameter and overall morphology, with each polymer.

The experimental setup and the relative concentrations of the different components were optimized by trying different concentrations and working distances and by checking the resulting material each time by SEM while gradually tuning the ES conditions.

The SEM images of the electrospun fibers obtained from the optimized solutions are compared in Figure 4, sections AI, BI, CI, for PVP, PEO and PVAc, respectively. For relative concentrations, refer to Table 1. A comparable fibrillar morphology was clearly observed: defined fibers, with homogeneous average diameters (375 ± 85 nm for PVP, 300 ± 69 nm for PEO, 560 ± 234 μm for PVAc), were obtained from solutions of different polymers. However, an effect of the nature of the polymer on the resulting diameter can be assumed, basing on the following trend: PEO < PVP << PVAc.

### 3.2. Thermal Treatments

**Figure 1:**
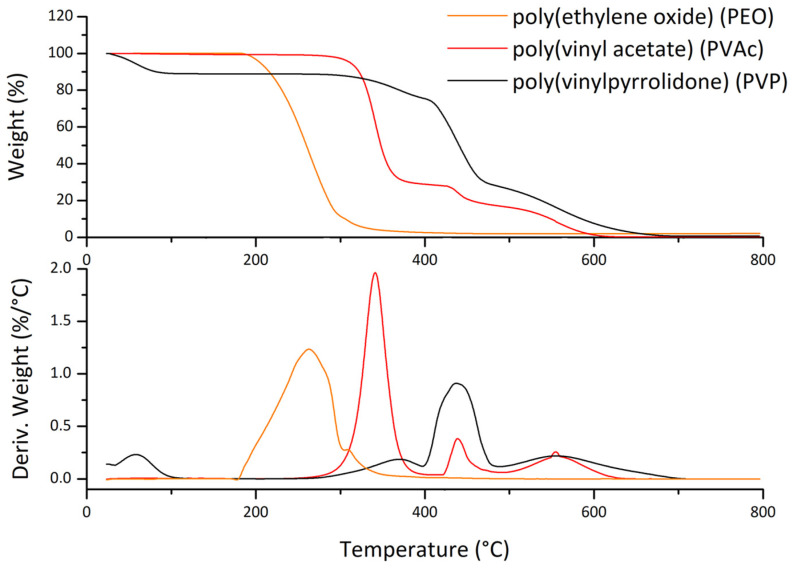
Comparison of thermo-gravimetric analysis (TGA) and derivative TGA (DTGA) of the different polymers in air, from RT to 800 °C, 10 °C/min.

In order to obtain the tin oxide, it is necessary to achieve the complete volatilization of the polymer and the degradation of SnO_2_ organic precursor [12,31]. In Figure 1, the TGA and derivative TGA (DTGA) of the sole polymer electrospun fibers is reported.

The degradation process starts at different temperatures for each polymer. The PVP shows an initial weight loss at 100 °C, mainly due to the presence of water (the high hygroscopicity of PVP is enhanced by the high surface area of micro/nanofibers); the thermal degradation starts at about 300 °C with the maximum rate of weight loss at about 450 °C. The principal mechanism during degradation of PVP is the depolymerization with the volatilization of vinylpyrrolidone, but as demonstrated by Lorìa-Bastarrachea et al., the presence of simultaneous reactions as the fragmentation of polymeric main chain is noticeable [27]. For what concern the PEO degradation, it proceeds with the random chain scission of the C–O bond [32]: indeed the TGA shows a single step of degradation starting at about 180 °C with the maximum rate of weight loss at about 280 °C, a much lower temperature than the PVP. The PVAc thermal degradation is well-known and occurs through two-steps, i.e., the elimination of the acetate groups between 230 °C and 400 °C and the subsequent chain fragmentation [26]. All the polymers are completely volatilized through thermo-oxidation.

**Figure 2:**
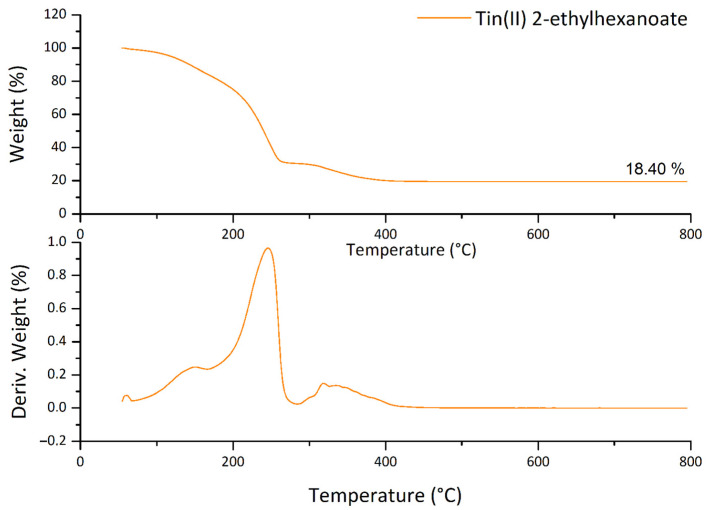
TGA and DTGA of the tin(II) 2-ethylhexanoate. The residue after partial volatilization and conversion in SnO_2_ was 18.40%.

The TGA analysis, in air, of the tin (II) 2-ethylhexanoate is shown in Figure 2.

The DTGA reveals at least three steps of weight loss, with the maxima centered at 150, 250 and 340 °C, respectively. A competition between the volatilization of SnEt (the boiling point at 296 °C) and the conversion into oxide is assumable; if the SnEt is completely converted, the residue should be around 34 wt % instead of the observed 18.4 wt %. Therefore, the precursor is not completely converted in the corresponding oxide, but it is partially lost by volatilization.

**Figure 3:**
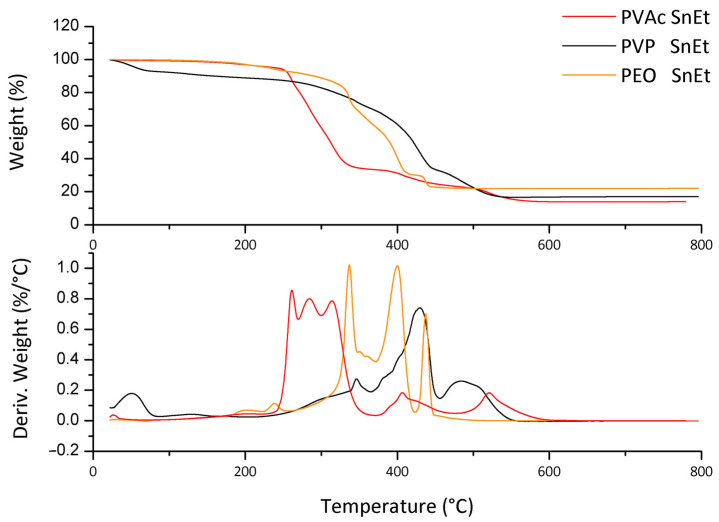
Comparison of TGA and DTGA obtained for different polymers containing precursor in air, from RT to 800 °C, 10 °C/min. The quantity of residue was 22% wt. for PEO, 17% wt. for PVP and 14% wt. for PVAc.

The TGA in the air of the electrospun fibers containing the oxide precursor is reported in Figure 3.

Most of the weight loss profile is related to the volatilization of the polymer matrix. Thermograms, however, show that the degradation proceeds at different temperatures if compared to the pure polymers: it is necessary to keep in mind that the weight/weight proportion between precursor and polymer is about 40/60. Therefore, the quantity of organic phase eliminated coming from the SnEt is relevant. Still, it is also true that hexanoate is a small molecule, especially when compared to polymer chains, whose evaporation/degradation starts at lower temperatures; hence it is also possible to hypothesize a sort of protective effect for the molecules trapped in the polymeric entanglement.

In all cases, however, more complex behavior and the formation of a residue stable at 800 °C is noticeable in the DTGA. Since all thermogravimetric analysis was driven in air, the residue should be the tin oxide. The expected quantity of residue, with a complete conversion of the precursor in SnO_2_, is 14.0%, identical for each polymer since the polymer/precursor ratio is the same. Nevertheless, the obtained quantities by TGA analyses are 22% wt. for PEO, 17% wt. for PVP and 14% wt. for PVAc. The value obtained for the PVP needs a correction because it is necessary to consider the initial weight loss related to the adsorbed water: the residue for PVP, after correction, is 18.20%. With the exception of PVAc, the residue is more than expected.

A synergistic effect in the formation of residue in the presence of polymer can be assumed. As seen before, the tin (II) 2-ethylhexanoate, if heated in the same conditions, volatilizes, leaving a small amount of residue. Therefore, the presence of the polymer prevents the volatilization of the precursor and leads to a higher quantity of residual material. Moreover, the yield in oxide is related to the polymer matrix; indeed, if we consider the PVAc, the yield is lower than the other polymers since the polymer starts its degradation at a relatively low temperature. The thermal oxidation of PEO at higher temperatures avoids the volatilization of the precursor and allows to obtain more oxide residue. For the PVP, there is an intermediate situation; the polyvinylpyrrolidone is a highly hygroscopic polymer and the quantity of residue, considering the presence of adsorbed water, is 19%, still lower than that obtained for the PEO. In this case, probably the removal of water can produce a stripping effect, subtracting part of the precursor.

Based on the results obtained by TG analyses, the suitable temperatures for calcining the electrospun samples were chosen, taking into account the temperatures at which the weight losses are concluded: 550, 450 and 600 °C were used for calcining the fiber mats obtained with PVP, PEO and PVAc, respectively.

**Figure 4:**
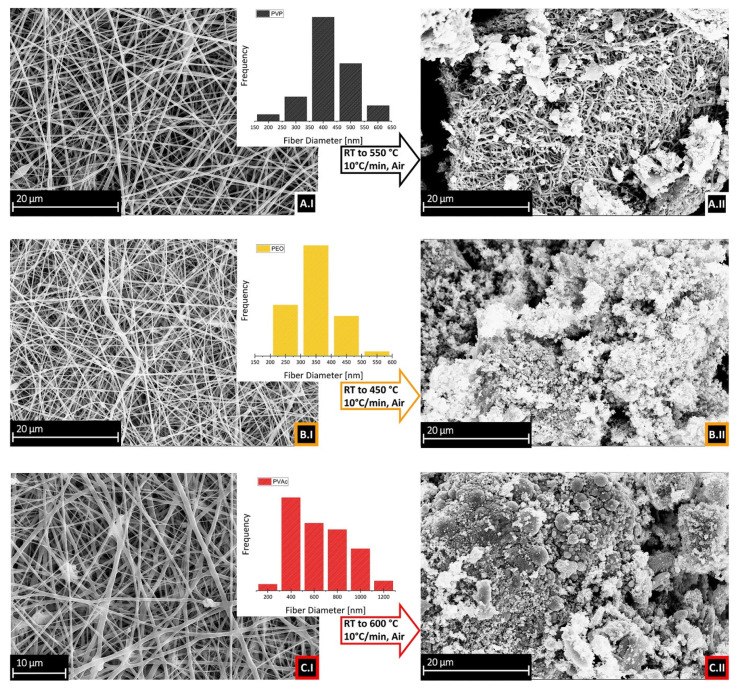
On the left: SEM images of the fibers obtained from different polymers: (**A.I**) PVP+ tin(II) 2-ethylhexanoate; (**B.I**) PEO+ tin(II) 2-ethylhexanoate; (**C.I**) PVAc+ tin(II) 2-ethylhexanoate. On the right: SEM images of SnO_2_ after calcination of fiber mats, from different template polymers: (**A.II**) PVP; (**B.II**) PEO; (**C.II**) PVAc. Insets: distributions of the diameters of the fibers shown in (**A.I**–**C.I)**. Instrumental magnification 2000×.

The morphology of the samples after the calcination in the oven is reported in Figure 4A.II,B.II,C.II. The fibrillar morphology observed before calcination (Figure 4A.I) is maintained in the sample with PVP (Figure 4A.II). Indeed, as seen in many cases [12,16], the PVP acts as a template for the formation of fibers. The situation is different for the oxide obtained from PEO and PVAc: the polymer matrix, after calcination, leaves a non-fibrillar morphology, different for each sample. In particular, for the PEO sample, a “flake-like” structure is clearly visible in the SEM image, Figure 4B.II. The PVAc sample, shown in Figure 4C.II, shows a structure of sintered “sphere-shaped” particles after thermal treatment.

The unique shape of the final oxide residue could be related to the different melting points of the template polymers. The approximate melting point is 150–180 °C for PVP, 66–75 °C for PEO and 58–60 °C for PVAc [33]. Melting points reported here refer to crystalline powders, so temperatures may vary in the presence of nanofibers, but it is evident a considerably higher melting point for PVP. Since PVP is the only polymer in this study that seems to retain the fibrillar morphology (as already found in our previous works [12,31]), it can be proposed that the formation of the oxide occurs before the melting of the fibers. Therefore, the polymer acts as a scaffold for the formation of a fibrillar structure. The same did not happen with the other two polymers, but the formation of nanoparticles, as shown in the next section, was not affected.

### 3.3. Characterization of Tin-Oxide

**Figure 5:**
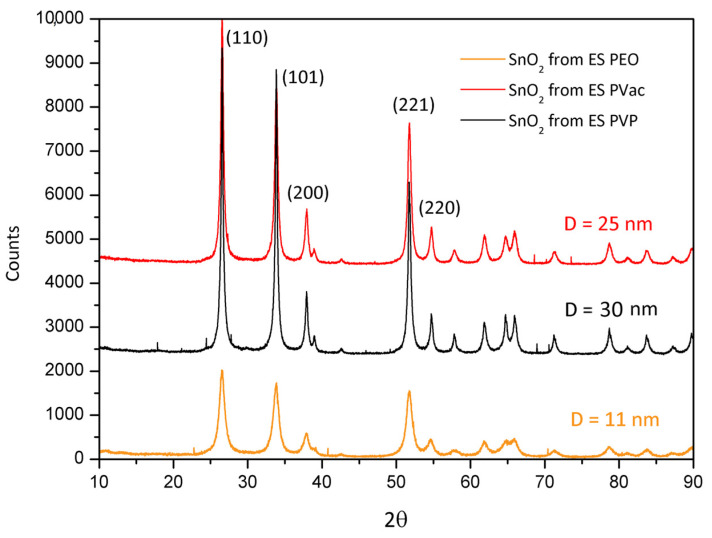
XRD patterns of SnO_2_ from different polymer templates.

The diffraction patterns of the three samples of SnO_2_ obtained after calcination, SnO_2_(PVP), SnO_2_(PEO) and SnO_2_(PVAc), are reported in Figure 5.

The XRD confirms that the three samples are constituted by crystalline cassiterite (SnO_2_ in the tetragonal crystal phase, JCPDS file number 00-001-0625) but with a different mean crystallite size. The calculated size (D, via Debye–Scherrer equation) is 25 nm for SnO_2_(PVAc), 30 nm for SnO_2_(PVP) and 11 nm for the SnO_2_(PEO) sample. The mean size was calculated using as reference the (110) peak. Basing on the XRD findings, it is evident that SnO_2_ fibers, flakes and spheres are constituted by nanoparticles.

**Figure 6:**
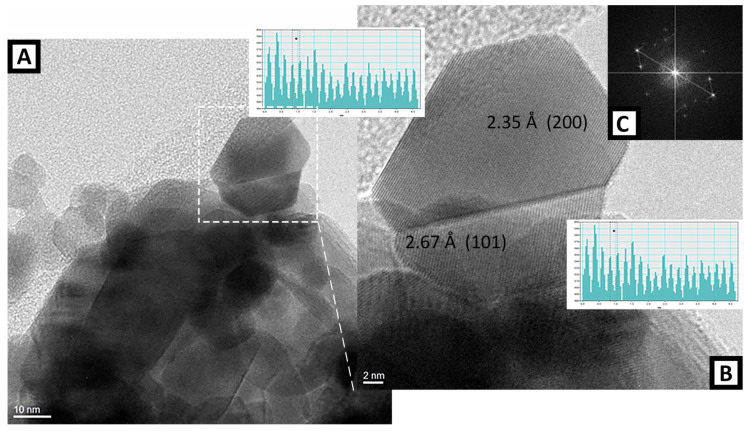
High-resolution transmission electron microscopy (HR-TEM) representative image of SnO_2_ from PVP/precursor fibers. Instrumental magnification 200,000×. (**B**) detail of a geminal crystal shown in (**A**). Instrumental magnification 500,000×. (**C**) Fourier-transform of the HR-TEM image is shown in (**B**).

**Figure 7:**
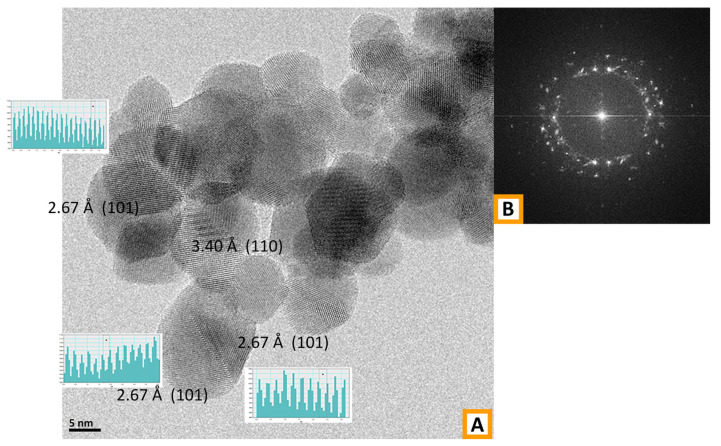
(**A**) HR-TEM representative image of the SnO_2_ sample obtained from PEO/precursor fibers and measures of the spacing between the diffraction fringes observed in different selected zones of the sample. Inset (**B**): Fourier-transform of the HR-TEM image in (**A**). Instrumental magnification 300,000×.

In order to obtain more reliable results in terms of particle size, HRTEM measures were performed on the SnO_2_(PVP) and SnO_2_(PEO) samples and the results are reported in Figure 6 and Figure 7, respectively.

The crystalline nature of both samples of SnO_2_ is highlighted by the presence of the diffraction fringes in the HR-TEM images: Figure 6 (B, detail of a geminal crystal) for PVP and Figure 7 (A, fringes observed in different particles) for PEO. For what concerns the SnO_2_ from the PVP sample, the analysis on the crystal in Figure 6B put in evidence spacing of 2.35 Å and 2.67 Å related to the (200) and (101) planes of cassiterite (JCPDS file number 00-001-0625). The corresponding particle size distribution appears broad, with a mean size is 18.7 nm with a standard deviation of 7.9 nm. In addition, the SnO_2_ particles obtained starting from the PVP sample appear quite heterogeneous in both size and shape since a considerable high number of big and squared particles has a diameter larger than 30 nm. As a consequence, even if most of the particles have a size in the 10–20 nm range, the average diameter is higher.

**Figure 8:**
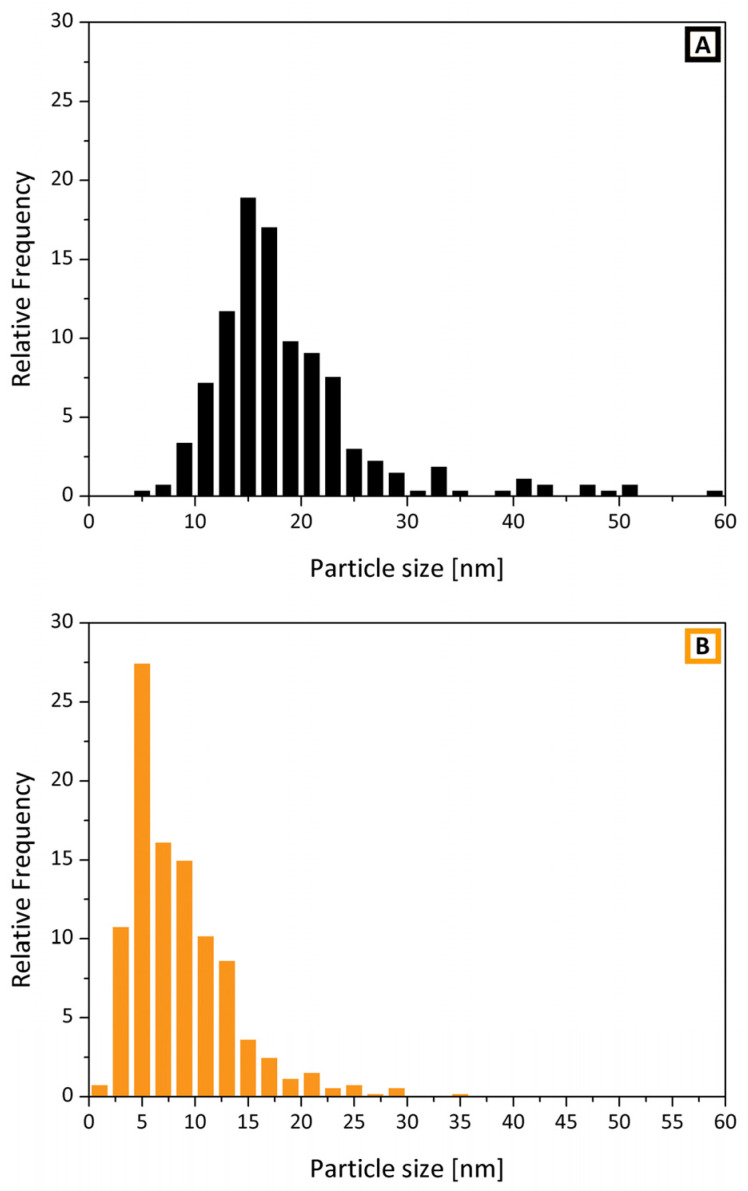
Comparison of particle size distributions of SnO_2_ nanocrystals obtained from (**A**) PVP (black, mean diameter = 18.7 ± 7.9 nm) and from (**B**) PEO (orange, mean diameter = 8.5 ± 4.9 nm).

Conversely, based on the particle size distribution (Figure 8B), the SnO_2_ nanoparticles obtained from PEO have a rounded shape with an average particle diameter of 8.5 ± 4.9 nm. It is worth noting that an appreciable number of particles show a diameter below 5 nm. The analysis of the diffraction fringes displayed by the SnO_2_ from the PEO sample evidenced spacings of 2.67 Å and 3.40 Å, related to (101) and (110) planes, confirming the presence of the cassiterite crystal phase (JCPDS file number 00-001-0625) also in this case.

It is worth noting that the different morphology exhibited by the two samples during calcination suggests that crystal growth is favored over nucleation in the case of SnO_2_ produced from PVP, while for the PEO samples, it appears that the nucleation rather than the growth is promoted.

**Figure 9:**
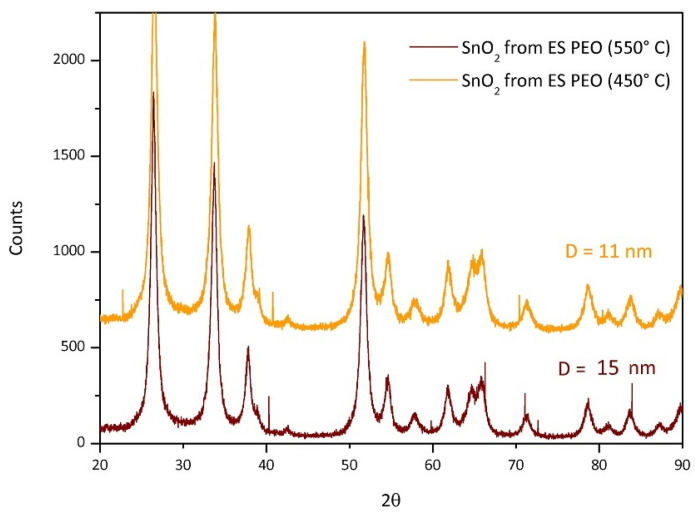
XRD patterns of SnO_2_ from PEO template calcinated at different temperatures: orange PEO 450 °C, dark red PEO 550 °C.

As previously evidenced, the calcination temperatures used are strictly related to the polymer volatilization in order to eliminate the organic phase. Hence, different temperatures were set for each polymer: 550 °C for SnO_2_ from ES PVP, 450 °C for SnO_2_ from ES PEO and 600 °C for SnO_2_ from ES PVAc. As seen, the suitable calcination temperature for PEO is considerably lower. To understand if the result is related to the temperature or to the templating polymer, an additional test was necessary. In Figure 9, the XRD patterns of two samples of tin oxide obtained from the same polymer, PEO, but calcined at 450 °C and at the same temperature of the PVP, i.e., 550 °C, are reported. The mean crystallite size of 15 nm is obtained for the sample calcined at 550 °C.

It is evident from the comparison of the mean crystallite sizes that the polymer matrix influences the dimension more than the temperature. Anyway, the temperature difference of 100 °C clearly shows the expected relation between crystal growth and temperature since SnO_2_ obtained at 550 °C is actually larger. The polymer clearly affects not only the final shape of the oxide but also the crystallite dimension.

## 4. Conclusions

SnO_2_ was successfully obtained from the calcination of fiber mats obtained via electrospinning of different polymeric solutions containing tin (II) 2-ethyl hexanoate. Fibers with comparable size, diameter, and overall morphology were obtained with each polymer, poly(vinylpyrrolidone), poly-(ethylene oxide) and poly-(vinyl acetate), via optimization of the experimental setup and the relative concentrations of the many components of the ES solutions.

Starting from a similar fibrillar morphology, it was possible to obtain different SnO_2_ morphology after calcination in the oven: surprisingly, the fibrillar morphology seen before calcination was only maintained in the sample with poly(vinylpyrrolidone), evidencing that PVP acts as a template for the formation of fibers.

Contrariwise, for the oxide from PEO and PVAc, the polymer matrix, after calcination, leaves a non-fibrillar morphology, different for each sample: for PEO SnO_2_, a “flake-like” structures are clearly visible in the SEM image, for PVAc SnO_2_, a structure of sintered “sphere-shaped” particles is shown.

XRD characterization confirms the presence of crystalline cassiterite (SnO_2_) but with different mean crystallite sizes and shapes depending on the nature of the polymer. The calculated size (via Debye–Scherrer equation) is 25 nm for SnO_2_(PVAc), 30 nm for SnO_2_(PVP) and 11 nm for the SnO_2_(PEO). Moreover, the comparison between the particle size distributions obtained from TEM characterization indicates that the SnO_2_ nanoparticles obtained from PEO exhibited a lower average size due to an appreciable number of particles with a diameter below 5 nm. This feature can be ascribed to the effect of the presence of PEO during calcination, which could favor nucleation rather than crystal growth during calcination. By applying different calcination temperatures on fiber mat obtained by the same polymer, i.e., PEO, the influence of polymer not only on the final shape of the oxide particles but also on the crystallite size was definitively demonstrated. Future directions of the present study are now focused on possible applications of the obtained SnO_2_: given the possibility to obtain quite small particles and to tune particle size and morphology, it would be interesting to note a possible impact in sensor applications, for which the materials produced with this method have already shown interesting results [8,31,34,35].

## Figures and Tables

**Table 1:** Relative concentration (wt/wt %) for polyvinyl pyrrolidone (PVP), polyethylene oxide (PEO) and polyvinyl acetate (PVAc) solutions.

Polymer	POL (wt %)	SnEt (wt %)	DMF (wt %)	SnEt/POL RATIO
Polyvinyl pyrrolidone (PVP)	15.3	11.0	73.6	40/60
Polyethylene oxide (PEO)	8.7	6.1	85.2	40/60
Polyvinyl acetate (PVAc)	15.4	10.8	73.9	40/60
